# Holy water and biomedicine: a descriptive study of active collaboration between religious traditional healers and biomedical psychiatry in Ethiopia

**DOI:** 10.1192/bjo.2021.56

**Published:** 2021-05-05

**Authors:** Yonas Baheretibeb, Dawit Wondimagegn, Samuel Law

**Affiliations:** Department of Psychiatry, School of Medicine, College of Health Sciences, Addis Ababa University, Ethiopia; Department of Psychiatry, School of Medicine, College of Health Sciences, Addis Ababa University, Ethiopia; Department of Psychiatry, Faculty of Medicine, University of Toronto, Canada

**Keywords:** Low- and middle-income countries, psychosocial interventions, anthropology, transcultural psychiatry, schizophrenia

## Abstract

**Background:**

Religious and traditional healers remain the main providers of mental healthcare in much of Africa. Collaboration between biomedical and traditional treatment modalities is an underutilised approach, with potential to scale up mental healthcare.

**Aims:**

To report the process and feasibility of establishing a collaboration between religious healers and psychiatrists in Addis Ababa, Ethiopia. To gain insight into the collaboration through studies of patient demographics, help-seeking patterns, nature of illness and receptivity of the project.

**Method:**

This case study describes the process and challenges in establishing a collaborative psychiatric clinic for patients who are simultaneously receiving treatment with holy water, including an examination of basic clinical records of 1888 patients over a 7-year period.

**Results:**

The collaboration is feasible and has been successfully implemented for 8 years. A majority (54%) of the clinic's patients were seeing biomedical services for the first time. Patients were brought in largely by families (54%); 26% were referred directly by priest healers. Most patients had severe mental illness, including schizophrenia (40%), substance misuse (24%) and mood disorders (30%). A vast majority (92.2%) of patients reported comfort in receiving treatment with holy water and prayers simultaneously with medication, and 73.6% believed their illness was caused by evil spirit possession.

**Conclusions:**

A cross-system collaborative model is a feasible and potentially valuable model to address biomedical resource limitations. Provider collaboration and mutual learning are ultimately beneficial to patients with severe mental illness. Open-minded acceptance of cultural benefits and strengths of traditional healing is a prerequisite. Further study on outcomes and implementation are warranted.

The World Health Organization (WHO) estimates that >80% of the population in Africa attend traditional healers for healthcare, and 40–60% of these are for mental illnesses.^1^ The high reliance on traditional healers, especially in the rural, low-resource settings that dominate the mostly low- and middle-income countries (LMICs) of Africa, make traditional healers the *de facto* backbone of the mental healthcare system.^2–4^ In Ethiopia, an LMIC country of >110 million,^5^ the most common mental illness treatment involves holy water, an Orthodox Christian approach believed to embody the spirit of Christ in its healing power.^6^ Holy water is used by people of Orthodox Christian faith (43.5% of the population)^7^ and the general population at large,^8^ as there is a largely shared cultural, cross-religious belief (i.e. illness explanatory model) that evil spirit possession is responsible for mental health issues, and holy water is used as an aid to exorcise such demons.^9^ Studies have shown that the majority of Ethiopians prefer traditional and alternative healing methods such as holy water, to biomedical services for their major illnesses, and particularly for mental compared with physical illnesses;^10,11^ up to 98% of first encounters in mental health help-seeking are with traditional practitioners, such as holy water healers.^12^

Like much of Africa, Ethiopia has a limited biomedical mental health infrastructure. The number of psychiatrists, for example, is at 0.08 per 100 000 people, far below those in middle-income countries like China (2.2), or high-income countries like Canada (14.7).^13^ For schizophrenia, the treatment gap for those who could have benefitted from biomedical treatment, but never received any, was 90%.^14^ For those who did eventually receive psychiatric care, the average ‘delay’ in help-seeking was around 38 weeks.^15^ Nevertheless, efforts to scale up mental health services in LMICs have shown some modest success. Examples include utilising and strengthening existing community treatment resources, such as rehabilitation programmes; using non-specialists to optimise basic care and case identification; task-shifting or task-sharing to expand front-line mental healthcare capacity; psychoeducation of families and advocating for prioritisation and funding.^16–20^ However, these solutions tend to be focused on the biomedical side alone, ignoring the other, parallel and often more popular and culturally accepted side of traditional healers. Another possible innovation for scaling up biomedical care may be collaboration between the two modalities.^21^ Yet, although many have advocated for this, examples of actual collaborations are rare. One study in Nigeria showed that providing traditional healers with basic training led to traditional healers recognising more symptoms of major mental illnesses, better understanding the aetiological concepts of mental illness, reducing the habit of beating their patients as a form of treatment, and increasing their collaborative outlook.^22^ More recent collaborative work with traditional healers in Kenya for patients with depression,^23–25^ and mental health-related task expansion with traditional birth attendants,^26^ have shown very positive and inspiring results, and have highlighted this important topic.^27,28^ The current study reports on a now well-established collaboration in Addis Ababa, Ethiopia, and focuses on the process for initiating a successful collaboration. It uses patient data to show that this approach may create a new pathway to treatment, in which biomedical and traditional belief systems can cooperate.

## Method

### Study design

This study describes the process that led to the creation of a collaborative clinic for patients receiving holy water treatment, and examines the available basic clinical records (including demographic, diagnosis and medication information) for patients who have attended this clinic over the past 8 years.

### Creation of a collaborative clinic

#### Study setting

Biomedical mental health practitioners from the Ethiopian Mental Health Society (EMHS, a non-governmental organisation) and the Department of Psychiatry at Addis Ababa University have worked in collaboration with traditional healers, including priests and patient attendants of St Michael's Church and St Mary's church (two closely located holy water treatment sites) in Addis Ababa, Ethiopia, for 8 years. The two holy water treatment sites form a community, with approximately 1000 patients; they reside in simple dwellings near the two churches. Both church sites have a natural supply of spring water that has been deemed holy through historically recorded healing miracles. At St Michael's, for example, the miracle involved a severely ill man, who appeared to suffer from a mood disorder, taking in the waters and recovering. Alongside spiritual cleansing through water, various forms of exorcism and religious-based counselling are also offered. The services are provided for free. Positive word of mouth, developed over years of working with people with mental stress, contributes to the enduring and strong cultural belief that treatment with holy water is useful.

#### Participant roles

The patients are individuals who are seeking or brought to the two sites for holy water treatment. They participate in regular (up to daily) treatments, often aided by their attendants. Rituals vary, and typically involve drinking, immersing or washing in the water. The water temperature can be cold for washing, especially for early morning rituals. Some of the patients appreciate treatment, but some resist and want to leave, and some refuse to carry out any of these rituals or even fight the priests. At times, treatment is involuntary and some patients are held in place by the attendants. In extreme cases, the patients are chained to their residence to prevent them escaping.

The Orthodox Christian priests performing holy water treatment are highly respected in Ethiopian society, a status achieved through the central position of the Church and their extensive studies of the bible, philosophy, music and the healing arts. They are inspired by St Paul, the apostle who was a physician, and believe that holy water is part of the sacrament, related to the fluids that flowed from the body of Christ when he was on the cross, symbolising purity and holding healing power. Their healing role is well-entrenched and almost never challenged (i.e. no government or public health policy would try to dissuade people from using holy water as treatment for any illness). The priests perform all holy water treatment-related activities, as well as chanting, blessing, praying and very light ceremonial smacking with a religious cloth, to symbolise exorcising of evil spirits. The priests involved in the project generally hold an open and accepting stance toward biomedicine, citing biblical teachings that unconditionally encourage people to seek help when ill.

Across Ethiopia, families provide the bulk of care for patients with mental illness, as there are no formal community mental health services. Families bring their ill members to holy water sites when they are overwhelmed; they find and pay attendants for services, and typically return to visit regularly; although for most, the residential treatment is a welcomed form of respite.

Attendants are personal care-takers, paid by the family, to look after the patients staying at holy water sites for the duration of their treatment. They negotiate housing and services for the families and patients, and provide multiple forms of care, including supervision of patients, accompanying patients during the holy water rituals, helping with prayers, and sometimes administering restraints for those involuntary patients at risk of escaping or for those patients who become violent. The attendants facilitate communication between the families, patients and priests.

The EMHS was established by families who have severely mentally ill family members. It provides support and advocates for those with similar struggles. As an Ethiopian community-based, non-governmental organisation sharing a common mission with the priests, the EMHS is a key partner of this project, facilitating communication and relationship-building between the priests and psychiatrists, and financing the bulk of medications.

Participating psychiatrists and residents are affiliated with the Department of Psychiatry at Addis Ababa University, which contributes staff time, some medications and academic guidance. The department is an academic and clinical leader in the region, with five affiliated teaching hospitals and a 3-year residency training programme with a capacity of 15 students per year.

#### Building mutual understanding and relationships via workshops

Initial meetings between the first author (Y.B.) and leaders of the two churches were facilitated by the EMHS and the headquarters of the Ethiopian Orthodox Church. As a first step, participants agreed to a series of five workshops and consultation rounds at each other's locations of practice. The first workshop was closely planned by the university faculty and the priests, and was hosted at the church, with invitations extended to all of the priests and attendants. About 100 people came, including 50 priests. It achieved the goal of introducing each group to the respective perspectives and understanding of mental illness in biomedical and traditional explanatory models. The atmosphere of the meeting was mutually respectful, participants shared lunch and established a working relationship. Booklets and education materials about severe mental illness written in Amharic, the national language, were shared.

The second consultative workshop took place at a nationally known teaching hospital, the Tukur Anbessa Specialist Hospital. The biomedical partners presented their scientific knowledge and treatment approaches in more detail. One of the highlights was a religious blessing conferred by an Orthodox Christian priest from the Church headquarters.

The remaining workshops focused on common clinical issues. The third looked at stigma, experienced as a common barrier for patients seeking help in both traditions. The highlight was acknowledging that, because holy water treatment was much more culturally accepted, it may be associated with lower stigma. The fourth workshop focused on abuses encountered by patients with mental ill health as part of their treatment and care, and the need for raising awareness of their struggles and an end to poor therapy practices. Both groups acknowledged challenges in managing involuntary patients who might be dangerous to themselves or others. Each group shared strategies that had been effective, and noted possible synergies. In the safe space of the workshop, some priests and attendants reflected that they regretted beating some patients out of frustration, and sought to change their practice; the psychiatrists also shared their versions of mistreatments in hospitals, and a wish to improve.

The fifth and final workshop allowed both parties to summarise their mutual learnings, and come to an agreement on a model for ongoing collaboration, in the form of a clinic in the holy water treatment community site. To promote the collaborative clinic, the project leads invited journalists to write stories of healing and to interview priests, attendants, psychiatrists and members of the EMHS for local radio shows and print media. Media coverage was positive for both general mental health awareness and the initiative.

#### Creating the clinic

The clinic was created close to the two churches that offered holy water treatment in 2012, with a mandate to welcome any patient seeking holy water treatment who was also interested in receiving biomedical consultations and possible treatments. Based on the relationship established in the workshops, priests and attendants began making referrals or assisted patients to attend the clinic. Notably, the church priests and personnel were not threatened by the biomedical model, and saw most activities as mutually beneficial. Building and maintaining this wider trust and rapport has been a crucial, ongoing priority for the biomedical practitioners.

The clinic operates once every 2 weeks, for 6–7 h. The team consists of a senior psychiatrist, a senior psychiatric resident, a clinical psychology student, a mental health nurse and an administrative clerk. The clinic also offers selected free medications, including essential antipsychotics and antidepressants that are widely available in Ethiopia, so treatment continuity is possible and costs are relatively sustainable. When patients leave the concurrent holy water and clinic treatment, a referral is made to their regional local health centre or hospital for continued care. The clinic has steadily grown in popularity, growing from an average of 15 patients per clinic day in 2012, to 39 in 2019.

### Understanding patients served by the clinic: an overview

Hard copies of the basic clinical records of all 1888 patients who attended the clinic between 2012 and 2019 were manually examined. The records contained limited sociodemographic and clinical data. Psychiatric diagnoses were reviewed and updated during the chart review, based on the DSM-5.

### Ethical approval

This research project involved retrospective, anonymous chart reviews, and presentation of aggregate data; no direct individual participant informed consent was sought. The methodology was approved by the Ethics Board of the School of Medicine, University of Addis Ababa.

## Results

### Demographics

Male patients outnumbered female patients by about four to one, and the majority of patients (82%) were aged 18–35 years. The vast majority (81.8%) of patients came from the city of Addis Ababa; the rest came from further afar, drawn by the reputation of the sites (within 125 km, and a few from further away). Most of the patients (80%) were single and educated (91% completed high school, which was much higher than the national rate, likely reflecting the urban status). Most patients (83.3%) were Orthodox Christians, 12.7% were Muslims and 4% were Protestant Christians. Almost all patients (98.7%) resided in the holy water treatment community; most stayed for less than a year (68%), typically 2–5 months, but 24% stayed for 1–5 years and 8% stayed for >5 years.

### Clinical diagnoses and duration of illness

The most common psychiatric disorder seen at the clinic was schizophrenia (40%), followed by substance use disorder (24%; mainly combinations of alcohol, qat, cigarettes and caffeine – different from the typical Western content of substance use disorder), bipolar disorder type 1 (18%), major depressive disorder (12%) and seizure disorders (6%). In terms of duration of illness before their visit to the clinic, 24% of patients reported a history of <1 year, 50% reported 1–10 years and 26% reported ≥11 years.

### Pathways of referral

More than a quarter (26%) of the clinic's patients were directly referred by priests from holy water sites or other churches; 13% were brought there by their holy water treatment attendants, often because their behaviour was disruptive or violent; and 7% came by themselves. Combined, 46% of the clinic's patients came from within the holy water treatment community, a brand-new source of referral since the creation of the clinic. The other 54% of the patients were brought to the clinic by family members. Although not specifically included on charts, it was observed that some of these patients were new to biomedical treatment, and others came because they sought help first at regional hospitals, but were turned away because of a lack of resources. Both groups were receptive to the availability of biomedical services alongside holy water treatment.

### History of mental health help-seeking and medication adherence

Regarding help-seeking pathways, 88% of the clinic's patients had their first-ever mental health contact at a non-biomedical source (faith-based prayer centres, general community traditional healers and holy water sites). By the time they came to the clinic, 48% of the patients had some form of previous contact with biomedical treatment but now came to holy water treatment instead. Of these, 89.2% had discontinued their biomedical medications; the main reasons were side-effects (55%), unaffordable costs (23%), a general refusal to take medication (10%) and a lack of improvement (7%). It was not feasible to track medication adherence rate at the clinic, but the general impression was a moderately high adherence, given the clinic's free medication, psychoeducation, supervision by the attendants and endorsement by the priests. Further, 52% of the clinic's patients had their first-ever contact with biomedical treatment through this very project. The general impression of the clinical and research staff was that the priests and attendants contributed greatly to these first encounters.

### Insight, explanatory model of illness and receptivity of collaborative treatment

Of those who received medications at the clinic, 71.9% had no awareness or insight to their mental illness. Most of these needed supervision by their attendants to take their medications. In terms of explanatory models, 73.6% of patients believed that they were possessed by evil spirits and trusted that the holy water would help exorcise those evil spirits. However, the vast majority of patients (92.2%) were comfortable in using holy water treatment and medication at the same time, and most patients (91%) swallowed their medications with holy water and performed their prayers before taking their medications. In terms of ongoing collaborative approaches, 41.3% of patients received one-to-one counselling from priests, 34.3% received psychoeducation and brief eclectic psychotherapy from the clinic, and 24.4% received counselling support from both.

## Discussion

There have been many calls-to-action to create more collaborative care to scale up mental healthcare,^29^ but few examples of what that looks like in practice, or models of how to achieve the goal.^30,31^ The current case study of combined holy water and biomedical clinic treatment provides a potential model for how a cross-system collaboration can work in practice, and tests strategies that emphasise mutual respect and learning. Our collaborative project has run for 8 years, and shows that people from a diverse social, religious, age and educational backgrounds are amenable to an innovative, dual-model approach in the treatment of their mental illness, with growing attendance.

Our findings highlight the sociocultural reality that the vast majority of patients chose traditional forms of healing over a biomedical approach when they made first contact in help-seeking. This is likely because of the high cultural acceptability and low social stigma associated with traditional treatment, as well as its relatively easy availability and low cost, a belief in the traditional explanatory models of mental distress, distrust of the biomedical model and family decisions on behalf of the patient.^25^ We also confirmed that biomedical mental health services remain in their nascent state in Ethiopia, even for those who accept such an approach and are geographically within reach of psychiatric care.

Many families wish to seek a residential form of care, likely because of high level of burden in caring for a family member with severe mental illness, and a need for respite and more intensive care.^32^ The clinic added to available support for a very ill population, most of them struggling with schizophrenia, addiction and bipolar disorder. Our study's preliminary data indicate that patients, families and attendants are accepting of biomedical treatment, and most importantly, that it received the endorsement and recommendation of the holy water priests. This is a reflection of the project's success, and is motivation to continue this valuable collaboration.

The engagement with the clinic did not significantly displace patients’ traditional views of mental illness or explanatory models. It is important to note that changing or displacing a culturally ingrained, and practically beneficial, low-stigma belief system is not a goal of the clinic. The goal is for new patients and referral sources to consider a combination model of treatment. The focus is on mutual respect and acceptance regarding the patients’ understanding of causality of mental illness, and based on these conditions, patients can feel comfortable ‘swallowing medication with holy water’.

When considering the barriers contributing to the relative scarcity of collaboration with traditional healers, there is a common belief among biomedical practitioners that the scientific model is superior, and a tendency to disregard traditional practices as irrational. There are also concerns that such a collaboration would have the appearance of condoning, or tacitly endorsing, certain practices that may be less consistent with ethical ideals, such as chaining patients or beating them – issues that came up as part of the consultation process.^33^ However, our study argues that such separation of the two models may result in missed opportunities to realise the considerable potential benefits of engagement. These include learning how traditional healers, often preferred by patients, connect with patients through a strong command of cultural understanding of the illness models and local knowledge of their needs and desires. These capacities are often undervalued in a biomedical approach, assuming that rational scientific thought is always superior.^34^ Also, among the core tenets of Ethiopian Orthodox Church, equality and a deep respect for the person are strongly emphasised by the priest healers, translating into an accepting and empathic attitude toward all patients. This ethical framework is consistent with Western ethics although certain practices may not align. It has been our observation that foundational respect and acceptance contribute to attenuating the stigma and discrimination experienced by the patients. This is worthy of reflection by both systems of practitioners.

At a conceptual level, this project has illustrated the possibility of garnering synergies from two seemingly opposing and irreconcilable practices. The merging of the two healing paradigms demonstrates the possibility of a complementary system of care.

### Limitations and next steps

The current study describes the clinic's creation, patients’ social demographic information and clinical diagnoses, results of the collaborative efforts, and the philosophical orientation and positive acceptance of such a model. It shows a promising example of cooperation between the two modalities; however, it regrettably lacks data in the basic clinical records to demonstrate effectiveness of the clinic. More outcome measures to show whether patients who attended the clinic had meaningful and sustained clinical improvement, or change in functioning level, would be ideal. The study is also limited by only having the perspective of the biomedical clinic, and the descriptive data has limited depth. We hope to address these limitations in more thoughtfully designed studies in the future. For example, comparisons between those who used the clinic and those who did not – in terms of changes in duration or course of illness, quality of life, functioning level, help-seeking, medication adherence, follow-up attendance and satisfaction level of the patient and their family – would be valuable. In addition, learning the strengths and weaknesses of the current model, and applying a quality improvement perspective to the collaborative model, would be useful. Qualitative understanding and reflections that inform further improvement of the collaboration among the priests, attendants, families and psychiatrists would be very desirable.

## Data Availability

The data that support the findings of this study are available from the corresponding author, Y.B., upon reasonable request.
